# Strychnia in Habitual Constipation

**Published:** 1862-07

**Authors:** E. H. Neyman

**Affiliations:** Cedar Bluffs, Iowa


### ARTICLE XXI.

### STRYCHNIA IN HABITUAL CONSTIPATION.

By E. H. NEYMAN, M.D., Cedar Bluffs, Iowa.

The cogency of circumstances render the occupation of many students, mechanics, and men of business and letters necessarily sedentary. It is here we find habitual constipates most frequently; to appreciate how frequently, it is only necessary to glance at the enormous quantity of patent pills vended by empirics, for the cure of this disorder. It is with the hope of correcting this wholesale use of cathartics that this communication is written.

*Etiology.*—It is only by a true understanding of the nature of disease that we can come to any rational mode of treatment. And rationalism—initiated by Descartes—must be the foundation of medicine. Without this, medicine ceases to rank as a science, for it ceases to have any sound or permanent basis. Constipation may arise from these causes:—

1st. Deficient contractility in the intestinal muscular coat.

2d. Deficient secretion; *a*, mucus, *b*, biliary.

3d. Obstruction of the passage of bile, by biliary calculi or other foreign matter, in the ductus communis cholidocus; by inflammation and œdema of intestinal orifice of bile duct; or by polypi.

Of these the second produces temporary but seldom, I conceive, permanent habitual constipation; the third is very rare; the first, then, must cover by far the greater number of cases. Let us take a brief peep into the region of pathology, and see what theory says in respect to this vast catharticism. Tonicity and irritability are the two inherent characteristics of muscular fibre. On this tonicity depends the vigor, and on the irritability its readiness, to react under various stimuli. What, in reference to these two functions, is the condition of the muscular coat of the intestine? Its tone and irritability are impaired; it fails to take cognizance of its natural stimulus, the feces. This lack of peristaltic action, constitutes a state of semi-paralysis. The property of healthy irritability has very narrow boundaries; if exhausted by over stimulation, recovery, under disease, is made still more doubtful. The vital stimuli, air, food, etc., renovate the tissues without exhaustion, but cathartics, while they may cause reaction, never directly increase vital force. If stimulation is the primary result, its good effects are lost in secondary sedation. If the intestine is stimulated into a renewed action, it subsequently relaxes into greater torpidity. These principles which regulate the action of medicines and pharmacological substances, are well understood by the profession. If, after the administration of a cathartic, we could remove the sedentary habits, substitute proper healthful exercise, pure air, laxative diet, etc., to raise the depressed vital power to a healthy standard, until the lagging recuperative powers could establish its permanency, all would be well. But this, in the greater number of cases, is impossible; the patient's avocation is sedentary, and on that he depends for his own and the sustenance of his family.

*Pathology.*—From what has been said under the head Etiology, it is clear, we think, that the pathological condition present, is deficiency in tone and irritability of the intestinal muscular coat.

*Symptoms.*—Deficient defecation, of course, is the certain index, but there are symptoms which are obscure. A. will complain that he is bilious; B. of dull, constant, and distressing cephalalgia; C. of an inability to apply himself closely to his studies; D. that he "aches all over," etc.; all of whom will apply for remedies for these symptoms, as one is more prominent than another, when, the fact is, all are suffering from the same disease.

*Prevention.*—"An ounce of prevention is worth a pound of cure." We must abstain from erroneous habits, use a vegetable diet, coarse bread, exercise in the open air, and above all, attend promptly to the calls for defecation.

*Treatment.*—Cathartics have been resorted to by the profession generally. To illustrate their deleterious effects we will suppose a case. A.'s habits are sedentary, there is engendered a torpidity of the intestine, which brings on a train of distressing symptoms; it wont do to depend upon nature, she is inadequate; healthful exercise is impossible; coarse diet insufficient; it wont do to wait—*rusticus expectat dum defluat amnis.* A physician is consulted, who either recommends some favorite patent nostrum—through convenience, or prescribes a dose of comp. cath. pills. The torpid and forgetful intestine is stimulated into an increased action, a copious evacuation is the result of its primary operation. The difficulty is corrected for the time, but the debility is increased, and it is soon found necessary to renew the stimulus, each successive dose multiplying the difficulty, and rendering the period between the doses shorter, so that at first it was only required simi-yearly, it becomes monthly, weekly, nay, every evacuation has to be solicited by some cathartic. So that, although it may be capable of unloading the intestine, and relieving the present difficulty, it but increases the cause.

If cathartics are so contra-indicated, what shall we say of laxative fruits, beverages, and aliment? These we believe to possess a different *modus operandi* from cathartics. Some act mechanically, others by being easy of digestion, still others by imparting tone to the gut. If some act similar to cathartics, their stimulation is of minor degree, as also their subsequent depression, hence are infinitely preferable to purgative medicines. From the pathological condition pointed, if we wish to perfect a cure, we must seek some agent whose action aims to elevate depressed irritability, gradually and slowly to the proper standard, imitating and aiding the natural recuperative powers, and characterized by permanency of action, like the vital stimuli. Something that will relieve the state of semi-paralysis. In reviewing the therapeutic effect of our various medicinal agents, we find none so capable of filling this indication as strychnia; and experience has demonstrated to me that its practical benefits are equal to its theoretical convenience. This substance appears to act principally on the *ganglionic system of nerves*, and the spinal column as high up, according to Flourens, as the medulla oblongata. To illustrate its applicability in derangement of the digestive function, let us take dispepsia as an example, a disease which, like habitual constipation, is plainly dependant on a weakened condition of the digestive organs. Their action in nutrition and assimulation owes their nervous influence to the *ganglionic system of nerves*, the system particularly under the influence of the various species of the strychnos. The extended sympathy of the digestive apparatus necessarily radiates an influence over the whole economy, causing general weakness, uneasiness, and a host of highly distressing symptoms. The stomach and intestine are intimately connected with the great solar ganglion of the ganglionic system; hence, if we derange these organs, we destroy the equilibrium of function, and deaden, more or less, the action of this entire system of nerves. Says a recent writer, in reference to the influence of the strychnos: "It has a tonic and stimulating influence over all the organs under the influence of the ganglionic system of nerves, by acting directly upon them, and exciting and equalizing their weakened action, and consequently restoring to equilibrium the digestive functions." My attention was first called to it by Dr. N. S. DAVIS, as a remedy, who spoke of it very highly in his lectures. I was at that time, a student of medicine, my habits sedentary, my studies requiring too much time to give myself proper exercise. I was habitually constipated; suffering from all its dreadful consequences. Like the rest of the world, I resorted to cathartics, which afforded me temporary relief, but left me in a condition worse than ever. Convinced that such treatment was inadequate to a cure, if not absolutely injurious, I was very readily induced to try Dr. DAVIS'S suggestion, and was very much gratified by the result, which was a complete cure. The formula recommended by Dr. DAVIS, and since used by me, is:—℞. Ex. hyosciamus,
Ferri sulphas, aa. gr. xxx.
Pulvis aloes,
Ex. nux. Vomica al. aa. gr. x.

M. Divide into thirty pills, one to be taken before each meal. This treatment to be persisted in until the constipation is overcome, and complete and regular defecation established. As adjuncts, I recommend the patient to exercise in the open air, as much as possible; to confine himself to a coarse diet; to attend promptly to all calls for defecation, and to make diurnal efforts to defecate. No case, ever so obstinate and distressing, but what has been promptly overcome by this treatment. Towards the completion of the cure, I modify my treatment by lengthening the interval between pills. The two cases, I was induced from what I conceived to be the pathology of the disease, and peculiar condition belonging to the individual case, to try the influence of electricity, by passing currents from the umbilicus to the coccyx, from one illium to the other, etc. The first one, was that of a shoemaker, aged 40. I passed the electrical currents for several times, without any manifest improvement. I then combined the strychnia, and imagined the improvement to be more rapid and marked than usual. This experiment was obscure, non-conclusive, and unsatisfactory. The second case, was complicated by paralysis of the bladder, to which the treatment was more particularly directed. Here, also, the two treatments were combined. By the use of catheter and persistent employment of the remedies, the patient's condition was gradually improving. This patient does not live in my section, having been seen while on a visit to the western part of this State, where he resides. In a case of paralysis of the sphincter, with irritability of the muscular coat of the bladder, decided improvement followed the use of strychnia and belladonna. In a paraphlegic patient, under treatment with nux vomica, the bowels are perfectly regular, although before the use of it there was constant constipation.

Hoping strychnia may prove as valuable a remedy in other hands as my own, I close this imperfect article.

